# Suspension Training: A New Approach to Improve Muscle Strength, Mass, and Functional Performances in Older Adults?

**DOI:** 10.3389/fphys.2019.01576

**Published:** 2020-01-10

**Authors:** Vitor Angleri, Samuel Domingos Soligon, Deivid Gomes da Silva, João Guilherme Almeida Bergamasco, Cleiton Augusto Libardi

**Affiliations:** MUSCULAB - Laboratory of Neuromuscular Adaptations to Resistance Training, Department of Physical Education, Federal University of São Carlos, São Carlos, Brazil

**Keywords:** suspension training, muscle strength, muscle mass, functional performance improvement, older adults

## Introduction

After the fifth decade of life, the natural aging process leads to annual reductions of 1.5% and 1–2% in muscle strength and mass, respectively (Morley et al., 2014; Zembron-Łacny et al., 2014). Additionally, muscle power is also reduced by 3–4% annually in older subjects (Macaluso and De Vito, 2004). These changes in the neuromuscular system are associated with impairments in functional performance (e.g., motor performance, control, balance, and mobility), increasing morbidity and mortality risks (Frontera et al., 2008; Cruz-Jentoft et al., 2010; Morley et al., 2014; Marty et al., 2017; McGlory et al., 2019).

Different resistance training (RT) modes have been proposed as effective interventions for enhancing muscle strength, mass, power, and functional performance in older adults [Chodzko-Zajko et al., 2009; American College of Sports Medicine position stand (ACSM), 2011]. However, less investigated RT modes also appear capable of optimizing these essential adaptations for older adults. Suspension training (ST) is a RT mode in which body segments are attached to suspended hanging straps, creating an unstable environment and using the body weight against gravity to perform multi-planar and multi-joint exercises (Byrne et al., 2014; Mok et al., 2015; Cugliari and Boccia, 2017). Interestingly, ST provides a progressive stimulus in the target muscles and a substantial activation in core muscles, suggesting it could be a promising RT mode to optimize muscle strength, mass, power, and functional performance (Maté-Muñoz et al., 2014; Ma et al., 2017).

The purpose of this manuscript is to provide rationale as to why ST could be a viable RT mode for increasing muscle strength, mass, power, and functional performance in older adults.

## Effects of Traditional Resistance Training on Muscle Strength, Mass, Power, and Functional Performance in Older Adults

Current guidelines widely recommend RT programs to mitigate age-related impairments in muscle strength, mass, power, and functional performance in older adults [Nelson et al., 2007; Chodzko-Zajko et al., 2009; American College of Sports Medicine position stand (ACSM), 2011]. In order to optimize muscle strength and mass gains in older adults, RT should be performed at least twice weekly, with 8–10 exercises for major muscle groups at moderate to high intensities and slow velocity (i.e., progressive RT [PRT]) (Nelson et al., 2007; Chodzko-Zajko et al., 2009; Fragala et al., 2019). In addition, if low loads are used, repetitions should be performed up to concentric failure in order to optimize adaptations (Van Roie et al., 2013). The PRT mode can also improve power and functional performance (Chodzko-Zajko et al., 2009; Churchward-Venne et al., 2015; Van Abbema et al., 2015; Fragala et al., 2019). However, these results are inconsistent (Skelton et al., 1995; de Vreede et al., 2005; Orr et al., 2008; Liu and Latham, 2009, 2011). For instance, two studies of Walker and colleagues showed that PRT [3 sets of 8–14 repetitions at 60–85% 1-RM (Walker et al., 2015) or 2–3 sets of 14–20 reps at ~50–60% 1-RM] (Walker et al., 2017) was effective in increasing muscle strength and mass but did not increase muscle power or functional performance (i.e., 7.5 m forward walk, 7.5 m backward walk, timed up-and-go test, and loaded 10-stair climb test) in older adults. Additionally, de Vreede et al. (2005) randomized 98 older women in a 12-week exercise program of functional-task exercise training (*n* = 33; 40 min of core exercises based on daily tasks) or PRT (*n* = 34; 3 sets of 10 repetitions with load progression). The remaining women were assigned to a control group (*n* = 31; kept their daily habits). Results showed improvements in functional performance only for the functional-task exercise group and muscle strength gains only for PRT. Furthermore, a meta-analysis with 121 trials (6,700 participants [>60 years]) showed that PRT resulted in a large and positive effect on muscle strength, while RT-induced effects on functional performance parameters ranged from small to large (Liu and Latham, 2009). For instance, the effects were moderate to large for chair rise, modest for gait speed (i.e., speed employed during the gait) and small and/or non-significant for balance and timed walk (i.e., time necessary to walk a set distance; Liu and Latham, 2009). Collectively, although PRT appears to be effective for enhancing muscle strength and mass, these gains may not be effectively transferred to power and functional performance (Orr et al., 2008; Granacher et al., 2013a; Fragala et al., 2019).

Regarding muscle power and functional performance improvements, recent guidelines recommend performing PRT in association with other RT modes specifically designed to this purpose within the same RT program (Chodzko-Zajko et al., 2009; Fragala et al., 2019). One RT mode deemed appropriate for this is power training (PT; i.e., using low to moderate intensities and maximal velocity; Chodzko-Zajko et al., 2009; Fragala et al., 2019). Fielding et al. (2002) compared the effects of PRT (3 sets of 8 reps at 70% 1-RM performed with 2 s for both concentric and eccentric actions) and PT (3 sets of 8 reps at 70% 1-RM performed at maximal velocity) on muscle power in older women. Results showed greater increases in muscle power for PT compared with PRT. Also, Ramírez-Campillo et al. (2014) showed higher increases in functional performance for PT (3 sets of 8 reps at 45–75% 1-RM performed at maximal velocity) compared with PRT (3 sets of 8 reps at 75% 1-RM performed with 3 s for each muscle action) in older women. Finally, Bottaro et al. (2007) demonstrated that PT (3 sets of 8–10 reps at 60% 1-RM performed at maximal velocity) produced higher increases in muscle power and functional performance compared with PRT (3 sets of 6–8 reps at 60% 1-RM performed with 2–3 s for each contraction) in older men. Considering that age-related impairments are greater in muscle power than muscle strength and mass, it is likely that PT could be a more specific RT mode compared to PRT for functional performance (Macaluso and De Vito, 2004; Petrella et al., 2004; Orr et al., 2008; Fragala et al., 2019).

Improving muscle power is not the only way to enhance functional performance. It has been suggested that the activation of the core muscles (i.e., muscle group composed of the abdominals, glutes, paraspinal, diaphragm, hip girdle, and pelvic floor; Gringmuth and Jackson, 2000) can play an important role in functional performance, as this muscle group allows power propagation to the body's extremities (Shinkle et al., 2012; Granacher et al., 2013a; Aguilera-Castells and Busca, 2018). Accordingly, a systematic review demonstrated that RT aimed at enhancing core muscle strength can be an alternative approach for balance/mobility and functional performance promotion in older adults (Granacher et al., 2013a). Additionally, the core muscles are responsible for balance maintenance and functional performance. It also allows daily life activities to be performed even on relatively unstable situations (e.g., during manipulation of unstable loads and/or walking on an irregular pavement; Behm and Anderson, 2006; Kibele and Behm, 2009; Behm et al., 2013; Granacher et al., 2013b; Aguilera-Castells and Busca, 2018). Therefore, considering the principle of training specificity, RT modes should attempt to simulate unstable environments when aiming to improve functional performance (Behm, 1995; Behm and Anderson, 2006; Aguilera-Castells and Busca, 2018).

## Suspension Training as a New Approach to Optimize Improvements in Muscle Strength, Mass, Power, and Functional Performance in Older Adults

**Figure 1:**
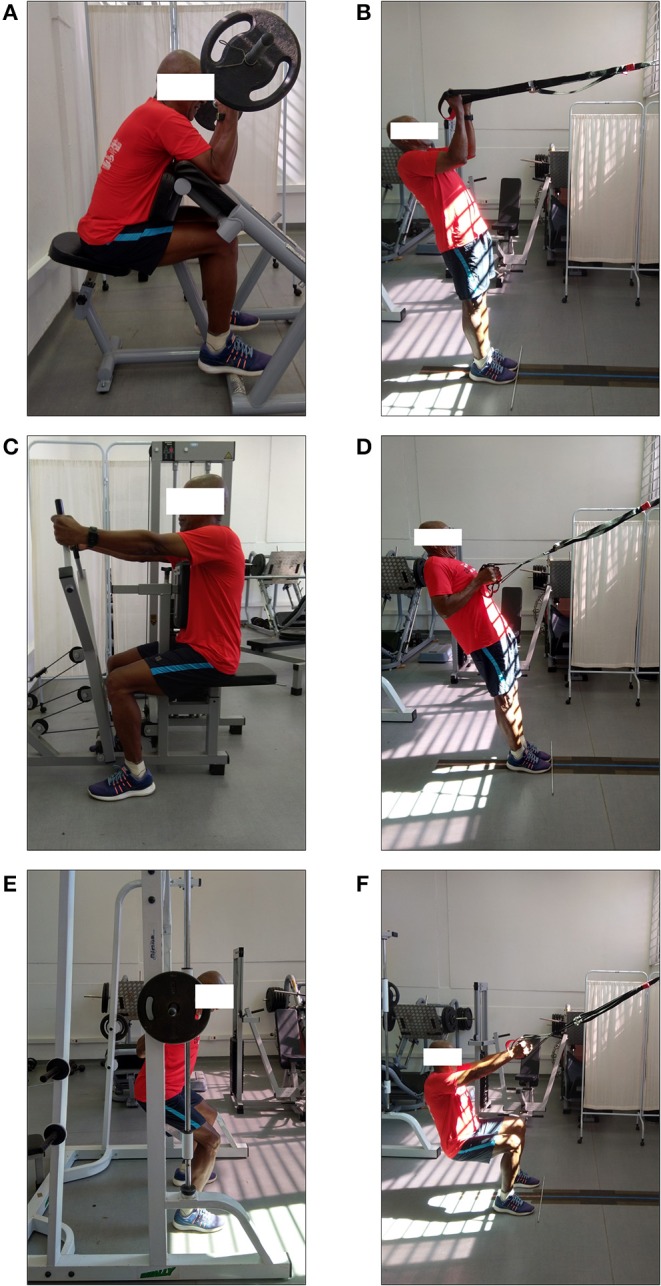
Exercise approaches in different training programs. Resistance training: elbow flexion **(A)**, seated row **(C)**, and squat **(E)**. Suspension training: elbow flexion **(B)**, seated row **(D)**, and squat **(F)**.

In the past years, strategies aiming to stimulate the core muscles through the simulation of an unstable training environment have been proposed (e.g., Balance Board^®^, BOSU^®^, and Swiss Ball) (Byrne et al., 2014; Cugliari and Boccia, 2017; Aguilera-Castells and Busca, 2018). In ST instance, instability is simulated by suspending the body's segments thought hanging straps, which creates an unstable environment where multi-planar and multi-joint exercises are performed using body weight and gravity as load agents (Byrne et al., 2014; Mok et al., 2015; Cugliari and Boccia, 2017). The ST is a RT mode that has a simple configuration, takes up little space, can be adjusted to the practitioners' requirements and permits a great variety of exercises (Figure 1). Although no study has investigated ST's safety, this method allows the adjustment and progression of the instability and training overload, even for frail elderly. Importantly, studies investigating the acute effects of ST support the notion that this RT mode may produce substantial improvements on functional performance due to a higher activation of the core muscles compared with RT modes performed on stable environment (e.g., PRT; Jiménez-García et al., 2019). In fact, Harris et al. (2017) compared the effects of push-up, inverted row, bridge and plank exercises performed in the PRT configuration (i.e., stable environment) with the same exercises performed using ST (i.e., unstable set-up) on trunk and core muscles activation in healthy adults. Results showed a higher activation of core muscles for ST compared with the stable PRT scheme. Additionally, a recent systematic review including 16 studies showed higher activation of core muscles for ST compared with PRT in the push-up, inverted row, prone bridge, and hamstring curl exercises (Aguilera-Castells and Busca, 2018). Regarding the chronic effects of ST, Ma et al. (2017) showed higher increases in core muscle strength and power for ST compared with PRT. Furthermore, Maté-Muñoz et al. (2014) compared the effects of PT and ST performed with the same exercises and maximal velocities. Results showed that ST is as effective as PT for improving the strength, power and movement velocity. However, these studies were performed with young (Maté-Muñoz et al., 2014) and trained cohorts (Ma et al., 2017). Therefore, future studies should investigate the effects of ST in older adults. Collectively, these studies allow us to suggest that the higher core activation inherent of ST, associated with its effectiveness in increasing muscle power, can make it a promising RT mode when aiming to improve functional performance in older adults.

Regarding muscle strength and mass gains, we (Barcelos et al., 2018; Nobrega et al., 2018; Damas et al., 2019) and other groups (Ahtiainen et al., 2005; Morton et al., 2016; Schoenfeld et al., 2016) have shown that exercising to concentric muscle failure produces similar increases in these neuromuscular adaptations despite different training variables or modes' manipulation. A recent study of Damas et al. (2019) reported similar muscle hypertrophy between a standard PRT and a protocol that systematically manipulated RT variables (i.e., exercise load, number of repetitions, type of muscle contraction, and inter set rest interval), both performed to concentric failure in resistance-trained men. Accordingly, other studies showed similar increases in muscle strength and mass in healthy adults when both bench press and push-ups (performed with body weight, likewise ST) were performed to concentric failure (Calatayud et al., 2015; Kikuchi and Nakazato, 2017). Hence, current literature points toward similar neuromuscular adaptations when exercising to concentric muscle failure, regardless of RT mode or scheme utilized (Burd et al., 2010; Damas et al., 2019). Although this hypothesis should be tested in the older population, evidence suggest that both traditional RT modes (e.g., PRT and PT) and ST would promote similar increases in muscle strength and mass when performed up to concentric muscle failure. If these hypotheses are confirmed, ST would therefore be a viable approach to produce improvements in muscle strength, mass, muscle power, and enhance functional performance.

## Conclusion

In summary, different RT modes such as PRT and PT could be used within the same RT program to improve muscle strength, mass, power, and functional performance. Additionally, the literature allows us to hypothesize that ST would be an alternative RT mode capable of increasing muscle strength, mass, power and functional performance due to the high activation of the core muscles induced by the instability, especially if sets are performed up to concentric failure. Finally, future studies should investigate the effects of ST on these outcomes in older adults. To investigate this paradigm, studies should measure muscle strength, mass outcomes, and functional performance through a battery of functional tests (e.g., habitual and maximal gait speed, chair stand, timed up-and-go and rate of torque development).

## Author Contributions

All authors contributed to the development of the manuscript, reviewed it, and approved the content of the final version.

### Conflict of Interest

The authors declare that the research was conducted in the absence of any commercial or financial relationships that could be construed as a potential conflict of interest.
